# Medical News and Miscellany

**Published:** 1897-08

**Authors:** 


### Medical News and Miscellany.

---

**Dr. T. W. Allen,** of Fort Davis, Texas, died May, 1897.

---

**Dr. W. T. Baker** has removed from Handley to Midlothian, Texas.

---

**Dr. A. A. Blassingame** has removed from Elmont to Sherman, Texas.

**Dr. M. L. Haggard,** of Midlothian, Texas, died June, 1897.

---

**Dr. Joe D. Becton** has removed from McKinney to Greenville, Texas.

---

**Dr. Thos. D. Wooten,** of Austin, has gone to Colorado, to spend the summer.

---

**Dr. T. H. Bragg,** a homeopathic physician, and former resident of Austin, died in Guadalajara, Mexico, on June 19th ult.

---

**Women to be Admitted.**—It is understood that hereafter women will be admitted as students into the medical classes of the College of Physicians and Surgeons of Chicago.

---

**A Dressing for Varicose Ulcers of the Leg.**—The *Progres Medical* attributes this formula to Simonelli:

Sodium chloride in impalpable powders..........10 parts
Powdered menthol........................................ 1 part
M. —*New York Medical Journal.*

---

**Attention** is called to Dr. Hopkins' advertisement in this issue, of Cock's Antibacilli Compound, a *rational* remedy for consumption. It is claimed for this remedy by those who have used it that 80 per cent. of cases of consumption are cured by it.

---

**Dr. J. S. Steele,** of Monterey, Mexico, oculist to the Saltillo division of the Mexican National Railroad, was married at Laredo, Texas, July 20th, ult., to Miss Agnes Clara Meehan, of that city.

The JOURNAL extends its congratulations and best wishes to its friend Steele and his bride. May they live long and prosper.

---

**Free Public Shower Baths in New York.**—At last the plans for the first permanent public baths have been completed, and the erection of the building will be at once commenced. It is to located on Rivington street, east of the Bowery, and will cost about $70,000. For sanitary reasons it was decided to have no swimming pool, but there will be an abundance of hot and cold shower baths. There will be accommodations for about two thousand persons a day, and the baths are to be kept open summer and winter. For those desiring to swim ample facilities are afforded by the fifteen free baths of the city, located at convenient points along the Hudson and East rivers, which are open during the summer months.—*Boston Medical and Surgical Journal.*

---

**Dr. W. A. Lockett,** of Brenham, Texas, surgeon of the Brenham Battalion of Light Artilery, T. V. G., was appointed Medical Director of Camp Culberson, and served in that capacity during the late State Encampment of the Texas Volunteer Guard at San Antonio, vice Dr. F. C. Ford, of Nacogdoches, who was detained at home by sickness.

---

**Excision as of Extensive Lupus with Skin Grafting.** —Matagne describes in the *Gaz. Med. de Liege*, his operation to relieve a girl of 16 of a lupus, 12 by 9 centimeters on the arm, which had developed fourteen years before, after vaccination with vaccine from another person. He tested the patient with tuberculin before attempting to operate, and completed the excision with transplantation of skin from the thigh. Five years have passed since without a relapse, and the patient seems absolutely cured.

---

**Thyroid Gland Extract as a Galactogogue.**—The *Inter-Colonial Medical Journal* of Australia, contains, among other papers, an interesting communication by Dr. R. R. Stawell upon this subject. The value of a reliable medicine that would promote the secretion of milk would be immense, so that Dr. Stawell's results will have an interest for many practioners. He details only nine cases, and, while admitting that this is but small clinical experience from which to generalize, concludes with some reservations that extract of thyroid gland is an efficient galactogogue. The dose given was from three to five tablets per diem. In view of Dr. Stawell's results we thlnk that medical men are justified in giving the treatment a trial.—*Exchange.*

---

**Osteopathy.**—The science of osteopathy is a combine of the old bone-setter's art, spiritualistic faith cure and Christian Science, constituting a method for the practice of charlatanry upon tne innocents who are credulous. With some neurasthenic and hysterical cases it is altogether probable that they have met with a degree of success in their methods of treatment. In some instances their massage work may do good by breaking up some old abnormal adhesions. That is all. Their methods of business are those of the charlatan, and quackish in the extreme. By their labors with the weak-minded they are likely to land some of them in hospitals for the insane. Their science is the science of imposition upon the credulity and nothing more.—*Cincinnati Lancet-Clinic.*

---

**Resignations.**—At the meeting of the Regents of the University of Texas, recently held at Galveston, the resignation of two of the professors in the Texas Medical College were received and accepted, to-wit:

Prof. A. G. Clopton, M. D., Physiology.

Prof. H. A. West, M. D., Practice of Medicine.

We have not learned the cause of these resignations. The vacant chairs will be filled, we learn, in time for the fall and winter course. Here now is a chance for the ambitious gentlemen of the profession. It is important that these chairs shall be filled by the very best teaching talent to be had, and the JOURNAL believes there are to be found *in Texas* men qualified for the positions.

---

**To Cut Short an Eruption of Herpes.**—In the *Journal des Practiciens*, for June 26, M. Leloir is cited as being of the opinion that it is possible to arrest the evolution of herpes by applying to the affected surface, as soon as the initial redness shows itself, a pledget of absorbent cotton soaked in a 1 to 50 alcoholic solution of resorcin, a 1 per cent. solution of thymol, a 3 per cent. solution of menthol, a 1 to 400 solution of carbolic acid, a 1 to 50 solution of tannin, or the following:

| | |
|---|---|
| Resorcin,...................................... | 3 parts |
| Cocaine,........................................ | 1 or2 parts |
| Alcohol,........................................ | 100 parts |
| M. | |

The cotton should be covered with an impermeable tissue to prevent evaporation.—*New York Medical Journal.*

---

**Mississippi Valley Medical Association.—Meeting to be Held at Louisville, October 5, 6, 7 and 8, 1897.**

The Executive Committee met recently at Louisville in conjunction with the local Committee of Arrangements, the following being present: Drs. Stucky, Grant, Mathews, Love, Holloway and Reynolds. It was determined to make the coming meeting the largest and best in the history of the Association, and everything points to a fulfillment of this endeavor. The railroads will make a round trip rate of one and a third fare, or probably one fare. The address on surgery will be delivered by Dr. J. B. Murphy, Chicago; the address on medicine by Dr. John V. Shoemaker, Philadelphia. Title of papers should be sent to Dr. H. W. Loeb, Secretary, St. Louis, Mo.

---

**The Internal Treatment of Pruritus.**—The *Gazette hebdomadaire de médecine et chirurgie* for May 30th attributes the following to Brocq and Jacquet:

1. Tincture of valerian, from two to fifteen drops a day.
2. Tincture of Belladonna, from five to six drops a day.
3. ℞ Ammonia valerianate...................... 1 part.
   Syrup of mint .............................. 20 parts.
   Linden water................................123 parts.

M. S.: From two to four spoonfuls a day [whether teaspoonfuls or tablespoonfuls is not specified.]

4. ℞ Extract of valerian........................$\frac{3}{4}$ of a grain.

   Powdered valerian........................a sufficiency.

M. To be made into a pill. S.: From two to eight such pills daily.—*New York Medical Journal.*

